# Pulsating Exophthalmos

**Published:** 1904-05-07

**Authors:** 


### Hospital Clinics and Medical Progress.

### PULSATING EXOPHTHALMOS.

Dr. F. W. Murray[1] states that pulsating exophthalmos is the result, in the great majority of cases, of an arterio-venous aneurysm of the internal carotid artery and cavernous sinus; in the remaining instances, which are extremely few, it is due to an aneurysm of the ophthalmic artery in the cranium. In about 71 per cent. of cases the condition is consequent upon traumatism, in the remaining 29 per cent. it owes its origin to chronic endarteritis of the internal carotid. Immediately following rupture of the artery the pressure in the cavernous sinus is raised, and interference is thus caused with the venous return through the ophthalmic veins. The symptoms of congestion may develop rapidly or slowly, according as the opening between the artery and sinus is large or small, and as a rule they appear early, but it is some days or weeks before they are fully developed. The characteristic symptoms are exophthalmos, pulsating tumour at the inner angle of the orbit, bruit, and pulsation. The most prominent sign is the protrusion of the eyeball, which in most cases reaches a high degree. The lids become swollen, œdematous, and discoloured, the conjunctiva is chemotic, and its veins are distended and tortuous. If paralysis of the ocular muscles be present, the axis of the eye deviates and motility is interfered with. Pulsation of the globe is marked, and in most cases is visible, but it can always be appreciated by palpation. The bruit is of a continuous character. It is increased at each systole, and is best heard over the globe, the pulsating tumour, and the supra-orbital region. The tumour at the inner angle of the orbit is composed of pulsating, tortuous, and distended tributaries of the superior ophthalmic vein. In the eye, the retinal veins are seen to be greatly distended and pulsating; hæmorrhages may occur, and in some cases papillo-retinitis is present. Sight may be unimpaired at first, but later it becomes diminished, and blindness may follow. Subjectively the symptoms are well marked, the patient complaining of severe headache, disturbing sounds in the head, and ringing in the ears, and it is commonly on account of these sensations that he seeks advice. Momentary digital compression of the carotid artery on the affected side is followed by the disappearance of pulsation and bruit, protrusion of the globe lessens, the pulsating tumour subsides, and the subjective symptoms are relieved; but, on removing the finger, the previous condition speedily returns.

The natural course of the disease, and the ultimate result, if left to nature, or if not affected by treatment, is a steady increase of the symptoms, when, having reached their height, the exophthalmos, the chemosis, and the swelling of the lids gradually subside, though the subjective symptoms and the pulsating tumour remain, and the eyesight, gradually growing weaker, is finally destroyed. In a few instances spontaneous recovery has resulted. To successfully treat the condition, a reduction or temporary abolition of the carotid pressure is necessary. This is usually accomplished by ligation of the common carotid artery. The chances of a cure by this operation depend upon whether the formation of a sufficiently firm clot will have occurred by the time collateral circulation becomes well established. Healing of the rent in the artery, in this manner, may be possible when the opening is very small or is due to disease of the arterial wall. The local conditions in the idiopathic cases are more favourable to thrombosis, while collateral circulation is not so rapidly or so freely established in these cases as in those where the vessels are in a healthy condition. The cure of traumatic cases, however, is hardly to be expected from ligature of one common carotid artery, since collateral circulation is so readily effected. In many of the reported successful results following ligation of the common carotid, the bruit which disappeared on tightening the ligature returned later on, while in others it never disappeared at all.

As spontaneous cure is possible, and because a few cases have recovered under medical treatment, some authorities still advise the trial of other measures before a resort is made to surgery. This Dr. Murray believes to be very unwise, since the chances of success are remote, and the time thus spent may result in the serious impairment, if not the loss, of sight. Digital compression of the common carotid may be tried, but the method, besides being painful, has other drawbacks, and is not likely to lead to a cure. Ligature of the common or the internal carotid is much preferable. Slomann, in 1898, reported 95 collected cases of pulsating exophthalmos treated by ligature of the carotid. The results were:—cured, 51 per cent.; improved, 17 per cent.; unimproved, 17 per cent.; died, 10 per cent. Whether the successes were or were not permanent is uncertain, as the majority of patients were lost sight of after their discharge from hospital. In a number of instances symptoms have reappeared after a considerable lapse of time, and in some of them ligature of the second common carotid has brought relief. In Dr. Murray's opinion ligature of the internal instead of the common carotid should be the operation of choice, as fewer channels for collateral circulation remain after the former procedure has been carried out.

[1] Annals of Surgery, March.

